# Physiological and pathophysiological role of ion channels and transporters in the colorectum and colorectal cancer

**DOI:** 10.1111/jcmm.15600

**Published:** 2020-07-13

**Authors:** Minglin Zhang, Taolang Li, Jiaxing Zhu, Biguang Tuo, Xuemei Liu

**Affiliations:** ^1^ Department of Gastroenterology Affiliated Hospital of Zunyi Medical University Zunyi China; ^2^ Digestive Disease Institute of Guizhou Province Zunyi China; ^3^ Department of Thyroid and Breast Surgery Affiliated Hospital of Zunyi Medical University Zunyi China

**Keywords:** colorectal cancer, colorectum, ion channels and transporters, physiological and pathophysiological role

## Abstract

The incidence of colorectal cancer has increased annually, and the pathogenesis of this disease requires further investigation. In normal colorectal tissues, ion channels and transporters maintain the water‐electrolyte balance and acid/base homeostasis. However, dysfunction of these ion channels and transporters leads to the development and progression of colorectal cancer. Therefore, this review focuses on the progress in understanding the roles of ion channels and transporters in the colorectum and in colorectal cancer, including aquaporins (AQPs), Cl^−^ channels, Cl^−^/${\text{HCO}}_{3}^{‐}$ exchangers, Na^+^/${\text{HCO}}_{3}^{‐}$ transporters and Na^+^/H^+^ exchangers. The goal of this review is to promote the identification of new targets for the treatment and prognosis of colorectal cancer.

## 1 INTRODUCTION

Colorectal cancer (CRC) is a common malignant tumour and a major health threat worldwide.^1^ Therefore, the elucidation of its aetiology and pathogenesis is important for the identification of effective therapeutic targets for early colorectal cancer diagnosis and prevention.

Ion channels and transporters are protein structures that exist in cell membranes and can mediate the transcellular transport of water and ions. These structures maintain the ion balance inside and outside of the membrane through the transmembrane transport of ions, causing some ions (K^+^, Na^+^, Ca^2+^, H^+^, ${\text{HCO}}_{3}^{‐}$ and Cl^−^) to be asymmetrically distributed on both sides of the membrane, thus forming an electrochemical gradient and maintaining the physiological functions of the organism. In normal colorectal mucosa, ion channels and transporters primarily regulate the transport of various ions, water‐electrolyte balance and acid‐base homeostasis thus to maintain the stability of the internal environment. However, the dysfunction of ion channels and transporters can lead to colorectal cancer, and to date, numerous studies have shown that changes in the status of ion channels and transporters are related to the proliferation, invasion, metastasis and apoptosis of colorectal cancer cells.^2^, ^3^, ^4^, ^5^ For instance, the down‐regulated expression of water channel proteins, also known as aquaporins (AQPs), in colorectal cancer cells was shown to result in decreased cell membrane permeability and migration.^4^, ^6^ Cl^−^ channels activate Wnt/β‐catenin signal pathway‐related proteins in SW480 and SW620 cells.^7^ Down‐regulation of Cl^−^/${\text{HCO}}_{3}^{‐}$ exchangers was shown to be associated with the progression of colon cancer,^8^ and the down‐regulation of Na^+^/${\text{HCO}}_{3}^{‐}$ transporters can reduce extracellular acidosis, cell proliferation, migration and invasion.^9^, ^10^ In human colon T84 cells, Na^+^/H^+^ exchangers induce ceramide production together with cisplatin (Diamminedichloroplatinum, DDP) to induce intracellular acidification, increase membrane fluidity and induce HT29 cell apoptosis.^11^, ^12^

**Figure 1:**
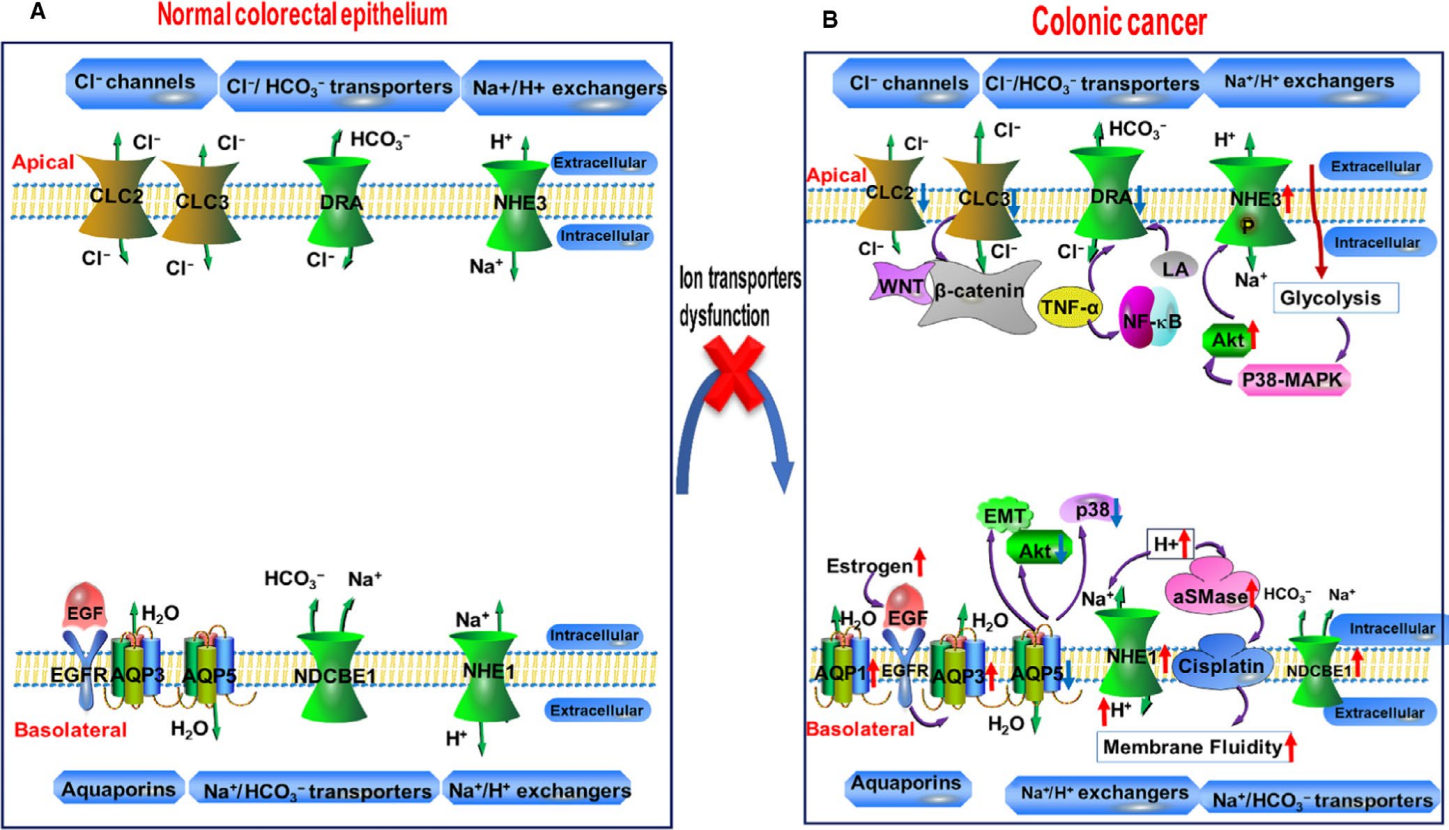
Schematic diagram of expression, location and function of ion channels and transporters in the normal colorectal epithelium and the alteration in the colorectal cancer. A, The intestinal cavity or circulating blood contains Na^+^, K^+^, ${\text{HCO}}_{3}^{‐}$, H^+^, Cl^−^ and H_2_O, and we summarize the transmembrane transport of some solute molecules through ion channels and transporters and their membrane localization in colorectal epithelial cells. B, During the process of colorectal development, the alteration of ion channels and transporters results in changes in ion permeability and distribution inside and outside the cell membrane as well as activation of various signalling pathways, providing a suitable microenvironment for the growth of tumour cells. The green arrow indicates the direction of flow in ion channels and transporters; the purple irregular arrow indicates a promoting effect; the red and blue arrows indicate the up‐regulation and down‐regulation of ion channels and transporters, respectively

Therefore, this review carefully summarizes the expression, localization and physiological role of ion channels and transporters, including the AQP water channel family, Cl^−^ channels, Cl^−^/${\text{HCO}}_{3}^{‐}$ transporters Na^+^/${\text{HCO}}_{3}^{‐}$ transporters and Na^+^/H^+^ exchangers in colorectal epithelial cells as well as their pathophysiological function in colorectal cancer (Figure 1, Table 1).

**Table 1:** Expression, localization and physiological and pathophysiological functions of ion channels and transporters in the normal colorectal epithelium and colorectal cancer

Name	Related channels/transporters	Human gene symbol	Localization	Physiological function in colorectal epithelium cells	Pathophysiological role in colorectal cancer
AQPs	AQP1	AQP1	Basolateral	Mediate water and small solute transport through the cell membrane	Inhibition of the AQP1 may block the migration and invasion of colon cancer cells^5^
	AQP3	AQP3	Basolateral	Permeable to glycerol and water	AQP3 promotes the proliferation and migration of colon cancer cells^28^
	AQP5	AQP5	Basolateral	Mediate water and small solute transport through the cell membrane	AQP5 induces the EMT process to enhance the migration and invasion of colorectal cancer cells^25^, ^31^
Cl^−^ channels	CLC‐2	CLCN2	Apical	Conducts Cl^−^ transport on the cell membrane	Regulated by phosphorylation^41^
	CLC‐3	CLC‐3	Apical	Conducts Cl^−^ transport on the cell membrane	Expressed in neuroendocrine colon cancer^44^, ^45^
Cl^−^/${\text{HCO}}_{3}^{‐}$ transporters	DRA	SLC26A3	Apical	Cl^−^ absorption, ${\text{HCO}}_{3}^{‐}$ secretion and absorption	DRA is down‐regulated in colon cancer^8^
Na^+^/${\text{HCO}}_{3}^{‐}$ transporters	NBCe1	SLC4A4	Basolateral	Na^+^ and ${\text{HCO}}_{3}^{‐}$ are co‐transported into cells	Silencing SLC4A4 reduces cell proliferation, migration and invasion^10^
Na^+^/H^+^ exchangers	NHE1	SLC9A1	Basolateral	Exchanging 1 intracellular H^+^ with 1 extracellular Na^+^	Inhibition of NHE1 may increase intracellular acidity and membrane fluidity^11^
	NHE3	SLC9A3	Apical	Na^+^‐H^+^ exchanger, pump out redundant H^+^ and pump in Na^+^ at the apical side	NHE3 is associated with the p38‐MAPK and PI3‐K signalling pathways^74^

## 2 AQP WATER CHANNELS

### 2.1 Functions of AQP water channels in the colorectum

Aquaporins comprise monomers composed of six alpha helix structural domains, and their formation is complete across the cell membrane to generate a pore and an assembly space for all four polymers, allowing cells to maintain their water balance through the cell membrane permeability gradient.^13^ Water channel proteins are present in many epithelial and endothelial cells as well as other tissues, where they function in cell regulation. Water transport plays an important role in water homeostasis.^14^ AQPs are classified into two categories according to their transport characteristics: classical water transport AQPs and solute transport AQPs. AQP1, AQP2, AQP4, AQP5 and AQP8 are considered to be aquaporins, which mediate the transport of water and small solutes through the cell membrane, while the glycerol‐porins AQP3, AQP6, AQP7 and AQP9 are permeable to glycerol and water.^15^ In addition, AQPs are involved in the transport of ammonia, urea, carbon dioxide, metals, nitric oxide and some ions.^16^ AQPs have an osmotic effect on anions, and the gated process of AQPs plays an important role in various processes, such as phosphorylation, pH, pressure, temperature and solute gradients.^17^

AQP1 is the first member of the AQP family and is a water‐selective transporter, which was first discovered in human erythrocytes and renal proximal tubules. In the gastrointestinal tract, AQP1 is expressed in the capillary endothelia of the human ileum and submucosa, but the expression of AQP1 is restricted to the mucosal microvascular endotheliu.^18^ At present, AQP1 has not been independently reported in normal colon tissue.

AQP3 is expressed in the basolateral plasma membranes of various human epithelial cells, such as those in the ileum,^19^ and distal colon,^19^ and is involved in the transport of water and glycerol in the distal colon.^20^ However, immunohistochemistry analysis has shown that AQP3 is primarily located in the basolateral membrane of epithelial cells in the lumens and crypt necks in the distal colon and rectum, being expressed less extensively in the crypt and barely expressed in the deep crypt.^21^ The results of immunocytochemistry and immunofluorescence analyses of the human colon have indicated that AQP3 is restricted to colonic villus epithelial cells and participates in water absorption in human colon surface cells.^22^

AQP5 is an aquaporin subtype and member of the aquaporin family that is located on human chromosome 12q3.^15^ AQP5 was first discovered in adult mouse and rat acinar cells^23^ and is expressed in a variety of epithelial cells, where its primary role is to regulate cell water homeostasis and growth signals. In addition, Matsuzaki et al^24^ showed that AQP5 is primarily distributed in the intercellular secretory tubules of cells secreting small salivary glands, pyloric glands and duodenal glands and plays an important role in water transport in these glands.

### 2.2 Effect of AQPs on tumour biological behaviours in the colorectal cancer

Recent evidence suggests that AQPs are associated with cell migration,^4^ angiogenesis^6^ and tumour growth^3^ and may therefore be involved in the occurrence and development of human cancers. AQP expression levels were shown to be up‐regulated in tumour cells of different origins, especially in invasive tumours, such as colorectal cancer.^6^ In studies of AQP1, AQP3 and AQP5 expression, some of these proteins were detected in colon cancer tissues and colorectal cancer cells, indicating that they play a role in colorectal cancer development. Furthermore, the results of in situ hybridization experiments revealed that AQP1 and AQP5 expression were induced in the early stage of disease and maintained throughout the late stage of colorectal cancer and metastasis to the liver, demonstrating their association with early colorectal cancer development.^25^ In addition, in colon cancer cases, Moon et al, observed that AQP1 was expressed in colorectal adenoma and primary and metastatic colon cancer but not in normal colon mucosa, suggesting that AQP1 plays an important role in the early development of colon cancer.^25^ Yoshida used a tissue microarray to demonstrate that some AQPs, such as AQP1, may be associated with colon cancer invasion. In this study, microarray analyses of patients with stage II and III colon cancer showed that the expression of AQP1 is associated with lymph node metastasis, lymphatic vessels and vascular invasion. A 5‐year survival analysis showed that the survival rate of patients with high AQP1 expression was significantly lower than of patients with low expression. In addition, according to multiple factor regression analysis, the expression of AQP1 is an independent poor prognostic factor.^26^ Furthermore, AQP1 expression in HT29 cells was shown to be inhibited by AqB013, and while high AQP1 expression effectively reduced cell migration and invasion, inhibition of its expression had no effect on the migration and invasion of HCT‐116 cells. Therefore, inhibition of the AQP1 water channel by AqB013 pharmacologically blocks the migration and invasion of colon cancer cells and can prevent the formation of endothelial cells in vitro. In the HT29 colon cancer cell line, the activity of AQP1 had no effect on inhibition of proliferation, whereas AQP1 activity decreased the proliferation rate of HCT‐116 cells by 17%.^5^ Therefore, AQP1 may be a promising biomarker for colon cancer prognosis.

AQP3 is expressed in colon cancer,^27^ and both epidermal growth factor (EGF) and oestrogen, which are known to be involved in the occurrence of cancer, are considered upstream regulators of AQP3 expression. After the addition of EGF to colorectal cancer cells in vitro,^27^ AQP3 expression was observed to be increased. Thus far, the mechanism by which the overexpression or ectopic expression of AQP3 accelerates tumour progression has remained unclear. Immunohistochemistry analysis of colorectal cancer cells revealed that AQP3 expression is correlated with the degree of differentiation, lymph node metastasis and distant metastasis of colon cancer. In cases with low levels of differentiation, lymph node metastasis and distant metastasis, AQP3 expression was significantly higher than that observed in cases with high levels of differentiation, no lymph node metastasis and no distant metastasis. AQP3 expression was shown to promote the proliferation and migration of colon cancer cells.^28^

AQP5 expression is up‐regulated in colon cancer, but its exact role in normal and cancer cells remains unclear, and increasing studies are being performed to elucidate how AQP5 induces tumorigenesis. AQP5 is expressed in early adenoma, late adenoma and adenocarcinoma but is barely expressed around normal colonic mucosa.^25^ AQP5 may be involved in the occurrence, development and metastasis of human colon cancer, and the increased expression of AQP5 in colon cancer tissues is related to tumour differentiation, tumour infiltration depth, lymph node metastasis and TNM stage.^29^ Furthermore, Shi et al assessed AQP5 mRNA and protein levels of in colon cancer tissue and then silenced AQP5 protein expression in HT29 cells. They observed that AQP5 inhibits HT29 cell proliferation and regulates the molecular mechanism of colon cancer cell resistance.

### 2.3 AQPs participates in the development and progression colorectal cancer via different signalling pathways

In addition, AQP3 and epidermal growth factor receptor (EGFR) are both involved in the differentiation and metastasis of colorectal cancer, thus serving as a potential inhibition target.^24^ In summary, in cultured cancer cells and the corresponding cancer tissues, increased AQP3 expression stimulates a variety of intracellular signalling pathways, leading to cell proliferation, migration, invasion and EMT.

AQP5 expression was shown to be significantly decreased after the expression of multidrug resistance (MDR) factors in HT‐29 cells, increasing the sensitivity to drugs and reducing the level of phosphorylated p38 lightning. However, the levels of AKT, phosphorylated ERK and phosphorylated JNK did not change. SB203580, a p38‐MAPK inhibitor, was used in a drug‐sensitivity experiment, the results of which showed that inhibition of p38‐MAPK can increase the drug sensitivity of 5‐FU and DDP in colon cancer cells. The above results indicated that p38‐MAPK is involved in the regulation of the drug resistance mechanism of HT‐29 cells.^30^ In addition, AQP5 overexpression leads to EMT in SW480 and HCT‐116 cells and decreases HT‐29 cell metastasis. In contrast, silencing AQP5 was shown to inhibit EMT in SW480 and HCT29 cells, and some studies have also shown that AQP5 can induce the EMT process to enhance the migration and invasion of colorectal cancer cells.^25^, ^31^

The results of the above studies have shown that the expression of AQP1, AQP3 and AQP5 is associated with tumorigenesis, proliferation, metastasis, reduced survival rates, lymph node metastasis, poor prognosis, cell migration and cell invasion.^32^ In addition, because blocking AQPs inhibits angiogenesis, tumour growth and metastasis,^32^ AQP may be a valuable biomarker for tumour diagnosis.

## 3 Cl^− ^CHANNELS

### 3.1 Physiological function of colorectal Cl^− ^channels

CLC proteins constitute a large family of chloride (Cl^−^) channel transport proteins that transport Cl^−^ and have multiple physiological functions.^33^ Most chloride channels are expressed on the cell membrane, while a few are expressed in organelles. The CLC channel performs Cl^−^ transport on the cell membrane to control the electrical activity of muscle cells and specific neurons, as well as the transport of liquids and electrolytes across epithelial cells and the acidification of intracellular vesicles.^34^

CLC‐2 is one of nine mammalian members of the CLC family. The CLC‐2 protein is encoded by the CLCN2 gene, which is located on chromosome 3q27.1 and encodes an 898‐amino acid protein, with a protein translation initiation codon located 100 bp upstream.^35^ CLC‐2 was first isolated in the rat heart and brain,^36^ and immunohistochemical studies of the colon indicate that CLC‐2 is primarily localized in the basolateral membrane of distal colonic cells in humans, rats, guinea pigs and mice.^37^ In addition, whole‐cell patch clamps and ultrasound imaging of the distal colon of guinea pigs have been performed. Laboratory studies have shown that Cd^2+^‐sensitive, hyperpolarized, inwardly rectified Cl^−^ conductors and Cd2^+^‐sensitive Cl^−^ conductors are present on the basolateral side of surface cells and epithelia, respectively,^38^ It is well known that the primary role of the colorectal water‐electrolyte balance is to absorb NaCl and water and mediate the transport of NaCl into colorectal cells through the apical Na^+^ channel and Na^+^/H^+^ and Cl^−^/${\text{HCO}}_{3}^{‐}$ exchangers. Therefore, CLC‐2 may be involved in transepithelial NaCl absorption on the surface of colorectal cells.

### 3.2 CLC family plays an important role to regulate colorectal cancer cell proliferation, differentiation, apoptosis and metastasis

Changes in CLC‐2 have recently been shown to lead to colorectal cancer.^39^ Human CLC‐2 was first cloned from the human epithelial cell line T84, and its predicted amino acid sequence is 93.9% identical to that of rat CLC‐2, having the function of generating a hyperpolarized activated Cl^−^ current.^40^ Using the patch clamp technique to detect hyperpolarized activated Cl^−^ currents in T84 cells, hyperpolarized activation of Cl^−^ and CLC‐2 channels in T84 cells was demonstrated to be regulated by phosphorylation^41^ but led to colorectal cancer, although the molecular mechanism underlying this phenomenon requires further study.

CLC‐3 acts as a Cl^−^/H^+^ transporter in the inner cell membrane,^42^ and in cancer studies, CLC‐3 has been shown to be involved in cell proliferation, apoptosis, metastasis and the cell cycle. CLC‐3 is expressed in both the ileum and colon, and its deficiency may be involved in the pathogenesis of inflammatory bowel disease (IBD) by promoting intestinal epithelial cell apoptosis and Paneth cell loss. Therefore, regulating CLC‐3 expression may be a novel IBD treatment strategy.^43^ In recent years, CLC‐3 has been shown to be expressed in neuroendocrine tumours such as human neuroendocrine colon cancer,^44^, ^45^ although little research on the role of CLC‐3 in colorectal cancer has been performed. In one study, after inhibiting the expression of CLC‐3, the expression of Wnt/β‐catenin signal pathway‐related proteins in SW480 and SW620 cells was decreased, indicating that CLC‐3 may be a potential treatment for colorectal cancer in the future.^7^

The role of chloride channels in tumorigenesis and development remains unclear. In recent years, chloride channels have been shown to be involved in cell proliferation, differentiation, metastasis and apoptosis (programmed cell death), and studying the effects of specific chloride ion channel subtypes on tumorigenesis may provide new methods and means for clinical targeted ion channel therapy.

## 4 Cl^−^/${\text{HCO}}_{3}^{‐}$ TRANSPORTERS

### 4.1 Physiological function of Cl^−^/${\text{HCO}}_{3}^{‐}$ transporters

Solute carrier 26 (SLC26) family members are anion/bicarbonate transporters that are expressed in the epithelial cells of multiple organs. Members of this family regulate homeostasis through the transport of various monovalent and divalent anions in epithelial membranes.^46^ SLC26A3 is a member of the SLC26A transporter family that is expressed in all epithelial cells to regulate Cl^−^ absorption and ${\text{HCO}}_{3}^{‐}$ secretion. In humans, the SLC26A3 gene is located on chromosome 7 and encodes a 764‐amino acid protein that is primarily expressed in the digestive system, especially in the duodenum and colon.^47^ SLC26A3 is primarily expressed on the cell membrane, inside the lumen, and chloride ion exchange occurs outside the lumen of the absorption chamber. Furthermore, the combination of the Cl^−^/${\text{HCO}}_{3}^{‐}$ exchanger SLC26A3 and the Na^+^/H^+^ exchanger regulates the absorption of most NaCl in the colon.^48^ Some researchers believe that the colon mediates the absorption of NaCl through the down‐regulation of the apical Na^+^/H^+^ exchanger‐3 (NHE3) and the Cl^−^/${\text{HCO}}_{3}^{‐}$ exchanger SLC26A3 (DRA). However, NHE3 is expressed at the highest levels in the proximal colon, with little or no expression in the distal colon, whereas DRA expression is absent in the early proximal colon and highest in the mid‐late proximal colon. Furthermore, DRA expression is prominent in the caecum, whereas NHE3 exhibits the opposite pattern, and one‐third of NHE3 and DRA is present in the colon. Mutation of the SLC26A3 gene results in an autosomal recessive disorder, congenital chloride diarrhoea (CLD), which enhances colonic proliferation and up‐regulation of ion transporters in the colon. CLD patients are at increased risks of IBD and colon cancer, indicating a new clinical role of SLC26A3 as a key molecule in colonic mucosal defence.^49^, ^50^ Furthermore, in a SLC26A3 knockout mouse model, the SLC26A3 gene was shown to potentially have two independent functions, as an anion transporter that primarily regulates Cl^−^/${\text{HCO}}_{3}^{‐}$ exchange in the large intestine and as an epithelial regulatory factor for cell proliferation. After SLC26A3 knockout, the mortality of mice increased, and the surviving mice showed CLD with weight loss and slow growth.^51^ In the colons of SLC26A3 knockout mice, the apical membrane chloride/base exchange activity decreased sharply, and the lumen content exhibited enhanced acidity.^51^ The results of the above studies suggest that SLC26A3 deletion leads to expansion of the colonic crypt epithelial proliferation zone, indicating that SLC26A3 plays an important role in colon tumorigenesis, although the specific molecular mechanism associated with this activity remains unclear.

### 4.2 Down‐regulation of SLC26A3 modulates colorectal cancer cell differentiation and progression

SLC26A3 was originally thought to be a transcription factor and a potential colon tumour suppressor based on protein libraries from normal colon and colon cancer cells.^52^ However, subsequent studies have shown that SLC26A3 encodes a membrane protein that specifically localizes to the tubular brush border (BB) membrane of differentiated colonic epithelial cells.^53^ While the mechanism of action of this protein in the normal colon is unclear, SLC26A3 mutation is known to lead to CLD, demonstrating that SLC26A3 is a transporter that plays a role in the colonic absorption of chloride.^54^ SLC26A3 was shown to be primarily expressed in colon cancer, but its expression is significantly decreased in colon cancer and colon cancer cell lines, and the down‐regulated expression of SLC26A3 was shown to be associated with the progression of colon cancer.^8^ Thus, down‐regulation of SLC26A3 in tumour cells may be a marker of differentiated colonic epithelial cells rather than a marker of cell proliferation.^55^

### 4.3 SLC26A3 alteration disrupts epithelial barrier function results in colorectal cancer via multiple signalling pathways

Some studies on the in vivo and in vitro mechanisms of SLC26A3 in the colonic epithelial barrier indicate that SLC26A3 directly binds to tight junction (TJ) proteins and affects their cellular expression. Knockout or overexpression of SLC26A3 leads to TJ protein and epithelial permeability, and TNF‐α treatment down‐regulates DRA and affects the integrity of the intestinal epithelial barrier by activating the NF‐кB signalling pathway. SLC26A3 overexpression can also partially reverse TNF‐α‐induced damage by stabilizing TJ proteins. This study suggests that SLC26A3 plays an important role in protecting the epithelial barrier and may provide therapeutic targets for the treatment of the intestinal environment in the future.^55^

## 5 Na^+^/${\text{HCO}}_{3}^{‐}$ TRANSPORTERS

### 5.1 Physiological function of Na^+^/${\text{HCO}}_{3}^{‐}$ transporters

The ${\text{HCO}}_{3}^{‐}$ transporter family includes Cl^−^/${\text{HCO}}_{3}^{‐}$ exchangers, Na^+^/${\text{HCO}}_{3}^{‐}$ transporters, K^+^/${\text{HCO}}_{3}^{‐}$ transporters and Na^+^‐dependent Cl^−^/${\text{HCO}}_{3}^{‐}$ transporters.^56^ However, this review primarily focuses on the physiological and pathophysiological effects of the solute carrier SLC4 family. This family consists of 10 members (SLC4A1‐5 and SLC4A7‐11), 9 of which encode ${\text{HCO}}_{3}^{‐}$ transporters and transport ${\text{HCO}}_{3}^{‐}$ through the plasma membrane. These proteins include three Na^+^‐independent Cl^−^/${\text{HCO}}_{3}^{‐}$ exchanger proteins (AE1, AE2 and AE3) and five Na^+^‐coupled ${\text{HCO}}_{3}^{‐}$ transporters (NBCe1, NBCe2, NBCn1, NBCn2 and NDCBE), where two of the Na^+^‐coupled ${\text{HCO}}_{3}^{‐}$ transporters (NBCe1, NBCe2) are electrogenic, while the other three Na^+^‐coupled ${\text{HCO}}_{3}^{‐}$ transporters and all three AEs are electrically neutral. Furthermore, the functions of SLC4A9 (AE4) and SLC4A11 (BTR1) are unclear, and most SLC4 members are inhibited by 4,4′‐diisothiocyanotoxine‐2,2′‐disulphonate (DIDS). The SLC4 ${\text{HCO}}_{3}^{‐}$ transporter plays an important role in regulating cell volume, CO_2_ transport, pH and electrolytes and has important physiological significance.^57^, ^58^

The Na^+^/${\text{HCO}}_{3}^{‐}$ cotransporter (NBCe1; SLC4A4) is one of five Na^+^‐coupled ${\text{HCO}}_{3}^{‐}$ transporters in the SLC family. After studying the proximal renal tubules in 1983, Boron et al described the first electro‐induced Na^+^/${\text{HCO}}_{3}^{‐}$ cotransporter.^59^ The NBCe1 protein is located in the basolateral membrane of the kidney proximal tubule,^60^ and in kidney tissue, NBCe1 mediates the movement of ${\text{HCO}}_{3}^{‐}$ plasma from the proximal tubule cells to the blood, thereby promoting the reabsorption of ${\text{HCO}}_{3}^{‐}$ from the lumen to the blood. The basolateral Na^+^‐H^+^ exchanger secretes H^+^ on the basolateral membrane, driving cytoplasmic CO_2_/${\text{HCO}}_{3}^{‐}$ equilibrium and promoting the formation of ${\text{HCO}}_{3}^{‐}$.^61^

### 5.2 Up‐regulation of NBCe1 promotes proliferation, migration and invasion in colorectal cancer

SLC4A4 (NBCe1) is expressed in tissues such as the duodenum, jejunum and colon and in colorectal adenocarcinoma cells.^9^, ^10^ In the colorectal adenocarcinoma LS174 cell line, mRNA expression of the Na^+^/${\text{HCO}}_{3}^{‐}$ cotransporter SLC4A4 was induced in an oxygen‐induced HIF1α‐dependent manner, indicating that the reversal of the Na^+^/${\text{HCO}}_{3}^{‐}$ exchanger is not used for tumour cell pHi regulation. Because silencing SLC4A4 reduces cell proliferation, migration and invasion and increases extracellular acidosis, SLC4A4 knockdown can reduce Na^+^/${\text{HCO}}_{3}^{‐}$‐dependent pHi recovery via partial extracellular acidosis.^10^ In SLC4A4 knockout and wild‐type mouse models, NBCe1 is active in the basal state, which may compensate for the acid load of the intestinal epithelial cells, and the loss of NBCe1 causes severe damage to Cl^−^ and liquid secretion reactions.^9^ In addition, inositol 1,4,5‐triphosphate receptor binding protein (IRBIT) increases NBCe1‐B and NBCe1‐C activity but does not increase NBCe1‐A activity.^62^ In summary, the molecular mechanism of SLC4A4 in the development of colorectal cancer is very important and requires further research to elucidate.

## 6 Na^+^/H^+^ EXCHANGERS

### 6.1 Expression, localization and function of Na^+^/H^+^ exchangers

The close coupling of the exchange of Na^+^ with H^+^ occurs on the membrane surfaces of almost all living cells. Na^+^/H^+^ exchangers (NHEs) are membrane transporters involved in a variety of physiological processes, including pH homeostasis, epithelial salt transport and volume regulation of systemic cells. In humans and other mammals, these transporters regulate the stability of the acid‐base balance inside and outside the cell by a 1:1 exchange of Na^+^ and H^+^ that is driven by the combined gradients of these ions. NHEs have different tissue expression patterns, cell localization patterns and physiological effects^63^ and are classified into three types according to their cell localization: serosa‐type NHEs (NHE1‐5/SLC9A1‐5), which selectively drive extracellular Na^+^ and cytoplasmic H^+^ electrical neutral exchange and work together to transport bicarbonates to regulate cell volume, fluid secretion, and electrolyte balance inside and outside the cell^63^, ^64^; endometrial or organelle‐type NHEs (NHE6‐9/SLC9A6‐9), which are present in most cells, primarily in intracellular regions although NHE8 is also expressed in the polarized apical membrane^63^, ^64^; and NHEDC1 and NHEDC2 (SLC9B1 and SLCB2), which are closely associated with the structure of fungal/plant NHA1 and bacterial NhaA Na^+^/H^+^ antivectors and have been reported to be expressed in the apical membrane of specific epithelial cells. Interestingly, NHE10 was recently shown to only be expressed in osteoclasts.^63^

The Na^+^/H^+^ exchanger‐1 NHE1 is a ubiquitously expressed integral membrane protein that was the first NHE subtype identified.^63^ NHE1 is located in the basolateral plasma membrane of most mammalian cell types, whereas NHE2 and NHE3 are present in the apical membrane and regulate electroneutral NaCl absorption, pHi and cell volume.^65^ NHE1 not only plays an important role in regulating pHi and cell volume, its activation also affects many downstream cellular events. Furthermore, NHE1 activity can prevent intracellular acidosis due to an excessive accumulation of H^+^ in cells, and although this process occurs in all cells, it especially occurs in proliferating and cancer cells. NHE1 is also a major factor involved in Na^+^ uptake and activity, and when combined with water and Cl^−^ uptake, it also controls cell shape and volume.^66^

NHE3 is expressed in the small intestine (duodenum, jejunum and ileum) and the colon^67^ and is responsible for most NaCl absorption in the intestines and kidneys, primarily in their proximal regions (the proximal renal tubules and small intestine are the primary sites of NaCl absorption). During this process, one molecule of Na^+^ and one molecule of Cl^−^ are absorbed together, resulting in no net charge movement.

### 6.2 NHE1 regulates colorectal cell apoptosis

NHE1 is activated by intracellular acidification in normal cells,^68^ but this process is dysregulated during carcinogenesis.^69^ In cancer cells, up‐regulation of NHE1 activity enhances H^+^ extrusion, leading to intracellular alkalization and the establishment of an extracellular acidic tumour microenvironment.^70^ NHE1 is involved in the development of tumours, playing roles in cell proliferation, cell migration, invasion, metastasis and apoptosis. The role of NHE1 in colorectal cancer and its mechanism, as well as the expression of Na^+^/H^+^ exchangers (NHE1, NHE2 and NHE4 subtypes) have been investigated in human colon T84 cells.^12^ DDP can trigger early ceramide production that is dependent on acid sphingomyelinase (aSMase), increase membrane fluidity, and induce apoptosis in HT29 cells, while ceramide significantly inhibits DDP‐induced apoptosis in the early stage. NHE1 has been shown to be inhibited on the cell membrane, resulting in increased intracellular acidity and aSMase activation. Furthermore, ceramide has been detected on the cell membrane, indicating that the DDP‐induced apoptosis pathway involves an H^+^‐dependent intracellular acidification of early NHE, resulting in aSMase activation and increased membrane fluidity.^11^

### 6.3 Up‐regulation of NHE3 results in the development of colorectal cancer via PI3‐K/AKT/ MAPK signalling pathway

In recent years, NHE3 has been studied in a variety of tumour tissues and cells, and the localization of NHE3 in tumour cells is consistent with that in normal tissues. PI3‐K, calcium/calmodulin‐dependent protein kinase II (CaM KII), PKA, PKG and PKC are involved in regulating the activity of NHE3, which binds to NHE3 via the BB PDZ protein of the NHERF family via specific receptors/signals via the activation of kinases at specific locations in the cell.^71^ Therefore, in NHE3‐expressing cells and most tissue models, the activity of basal NHE3 is regulated by PI3‐K, and PI3‐K inhibitors reduce the ion transport rate on the plasma membrane and NHE3 expression, especially in the rabbit ileal model. Because the absorption of NaCl did not change, this is the only example of PI3‐K not participating in the regulation of NHE3.^72^ In contrast, regarding the expression of NHE3 in Caco‐2 cells, wortmannin was shown to inhibit basal NHE3 activity by 50%.^73^ Furthermore, in colorectal cancer cell lines, D‐glucose activates Caco‐2 BB NHE3 through p38‐MAP kinase, MAPAKP2, PI3‐K and AKT2, and endothelin also affects the Ca^2+^‐sensitive kinase PYK2 in a dose‐dependent manner. To stimulate and inhibit NHE3, NHE3 must be phosphorylated, leading to NHE3 complex formation.^74^ These data suggest that NHE1 and NHE3 may be used as novel targets for the treatment of colorectal cancer, although further study is needed.

## 7 OPINIONS AND OUTLOOK

Ion channels and transporters play an important role in regulating the transport of colorectal transmembrane ions and in maintaining the balance of water and electrolytes. However, the mechanism by which water/electrolyte imbalance caused by defective ion transporter function leads to the development of colorectal cancer remains unclear. Thus, it is especially important to elucidate the aetiology and pathogenesis of colorectal cancer and to further explore novel tumour markers and therapeutic targets for early diagnosis and treatment. Therefore, in this review, we focused on the role of various ion channels and transporters in the regulation of normal colonic function and the development of colorectal cancer, including the AQP water channel family, Cl^−^ channels, Cl^−^/${\text{HCO}}_{3}^{‐}$ transporters, Na^+^/${\text{HCO}}_{3}^{‐}$ transporters and Na^+^/H^+^ exchangers. We hope to provide a basic, systematic summary of the description of this field to provide new directions for the prevention and treatment of colorectal cancer.

## CONFLICTS OF INTEREST

The authors declare no conflicts of interest.

## AUTHOR CONTRIBUTION

**Minglin Zhang:** Writing‐original draft (lead). **Taolang Li:** Funding acquisition (supporting); Writing‐review & editing (equal). **Jiaxing Zhu:** Investigation (equal). **Biguang Tuo:** Funding acquisition (equal); Supervision (equal); Writing‐review & editing (equal). **Xuemei Liu:** Funding acquisition (equal); Investigation (equal); Writing‐review & editing (equal).
